# The Zebrafish Orthologue of the Dyslexia Candidate Gene *DYX1C1* Is Essential for Cilia Growth and Function

**DOI:** 10.1371/journal.pone.0063123

**Published:** 2013-05-01

**Authors:** Gayathri Chandrasekar, Liselotte Vesterlund, Kjell Hultenby, Isabel Tapia-Páez, Juha Kere

**Affiliations:** 1 Center for Biosciences, Department of Biosciences and Nutrition, Karolinska Institutet, Huddinge, Sweden; 2 Clinical Research Center, Department of Laboratory Medicine, Karolinska Institutet, Stockholm, Sweden; 3 Folkhälsan Institute of Genetics, Helsinki and Molecular Neurology Program, Research Programs Unit, University of Helsinki, Helsinki, Finland; 4 Science for Life Laboratory, Karolinska Institutet, Stockholm, Sweden; Institute of Cellular and Organismic Biology, Taiwan

## Abstract

*DYX1C1,* a susceptibility gene for dyslexia, encodes a tetratricopeptide repeat domain containing protein that has been implicated in neuronal migration in rodent models. The developmental role of this gene remains unexplored. To understand the biological function(s) of zebrafish *dyx1c1* during embryonic development, we cloned the zebrafish *dyx1c1* and used morpholino-based knockdown strategy. Quantitative real-time PCR analysis revealed the presence of *dyx1c1* transcripts in embryos, early larval stages and in a wide range of adult tissues. Using mRNA in situ hybridization, we show here that *dyx1c1* is expressed in many ciliated tissues in zebrafish. Inhibition of *dyx1c1* produced pleiotropic phenotypes characteristically associated with cilia defects such as body curvature, hydrocephalus, situs inversus and kidney cysts. We also demonstrate that in *dyx1c1* morphants, cilia length is reduced in several organs including Kupffer’s vesicle, pronephros, spinal canal and olfactory placode. Furthermore, electron microscopic analysis of cilia in *dyx1c1* morphants revealed loss of both outer (ODA) and inner dynein arms (IDA) that have been shown to be required for cilia motility. Considering all these results, we propose an essential role for *dyx1c1* in cilia growth and function.

## Introduction

Dyslexia is the most common learning disability affecting approximately 5–10% of school children worldwide. The dyslexia susceptibility 1 candidate gene 1 (*DYX1C1*) is the first gene implicated as a candidate gene for dyslexia [1]. Although mixed replication results for *DYX1C1* in dyslexia were first reported, several independent studies that followed later have confirmed its association to dyslexia, verbal short term memory and orthographic skills in many populations [2], [3], [4], [5], [6], [7], [8], [9], [10].

Subtle malformations in the cerebral cortex have been associated with dyslexia [11], [12]. Consistent with the impairments seen in dyslectic brains, inhibition of *Dyx1c1* in rats by in utero RNAi was shown to affect neuronal migration in the neocortex resulting not only in similar cortical and hippocampal heterotopias but also impairments in the auditory processing, spatial learning and spatial working memory [13], [14], [15], [16].

Genetic studies in humans have led to the identification of several other candidate genes for dyslexia, among which *DCDC2*, *KIAA0319* and *ROBO1* have been strongly implicated in either neuronal migration in the developing cortex or axon and dendritic guidance [17], [18], [19], [20], [21]. In addition, analyses of brain structure and the SNPs in *DYX1C1*, *KIAA0319* and *DCDC2* in children and young adults have suggested a neuronal basis for reading abilities involving the white matter volume in the left temporo-parietal regions of the brain [22]. Despite the growing evidence that implicates defective cortical neuron migration in dyslexia, the precise cellular and molecular mechanisms are still not clearly understood. The DYX1C1 protein was recently implicated in regulating estrogen signalling [23].

The dyslexia candidate gene *DCDC2* was recently shown to localize to neuronal cilia upon overexpression and regulate cilia length and signalling [24]. The primary cilium, an organelle extending from the surface of the cell is present in almost all cells in vertebrates and is important for normal development and for various biological processes. Cilia are bound to the cell surface through the basal body and consist of a microtubule framework, termed the ciliary axoneme. The primary cilia or non-motile cilia have 9+0 axoneme, 9 outer microtubule doublets, no central microtubule and no ODA and IDA and mainly function as chemosensors or mechanosensors or osmosensors. Cilia on the epithelial cells of mammalian lungs and oviduct are motile and possess a 9+2 axoneme with two central microtubules and dynein arms [25]. Monocilia of mouse embryonic node are motile and generate a unidirectional fluid flow inside the node that is crucial for the initiation of organ left-right asymmetry [26], [27]. The importance of cilia in brain development and function has been recognized recently [28], [29]. Defects in cilia are associated with a broad range of human diseases categorized as ciliopathies, which share overlapping symptoms including neurological symptoms [30].

In this study we have used zebrafish as a model to study the biological function of *DYX1C1* through its orthologous gene *dyx1c1*. Here, we show that the zebrafish ortholog is expressed in ciliated organs including Kupffer’s vesicle (KV), otic vesicle, pronephros, spinal canal and olfactory placode. We performed a knockdown of *dyx1c1* using antisense morpholino oligonucleotides (MO) which resulted in pleiotropic phenotypes similar to those previously observed in zebrafish mutants with defective cilia [31]. Our results also show defects of cilia structure and length in various organs in *dyx1c1* morphants. Disruption of *dyx1c1* results in loss of ODA and IDA, thus disrupting cilia motility. Our results provide the first evidence that *dyx1c1* is essential for vertebrate ciliogenesis and function.

## Materials and Methods

### Ethics Statement

All experiments were carried out in accordance with ethical permits approved by the relevant ethical committee (Stockholm North Experimental Animal Committee Dnr N29-12). Dissection on adult fish was performed under tricaine treatment to ameliorate animal suffering.

### Zebrafish Maintenance

Zebrafish (Danio rerio) were reared and maintained according to standard procedures [32]. Wild-type embryos were obtained from AB strain fish by natural spawnings and raised at 28°C. Embryos were staged to hours post fertilization (hpf) and days post fertilization (dpf). To prevent pigmentation in embryos older than 24 hpf, phenylthiourea was used as described previously [32].

### Cloning of the *dyx1c1* mRNA

RNA was extracted from 50 zebrafish embryos at 50% epiboly stage using trireagent (Sigma Aldrich, St Lois, MO, USA). One microgram of total RNA was used for cDNA synthesis according to the Superscript III protocol (Invitrogen, Inc, CA). A proofreading polymerase Accuprime Pfx, (Invitrogen, Inc, CA) was used to amplify the coding region of zebrafish *dyx1c1* transcript. In short, one microliter of the cDNA synthesis reaction was used as template in a touchdown PCR ranging in annealing temperatures from 60°C to 56°C. Primers used were 5′-CGCTGAGGAGAGTCAGAGATG-3′ (forward) and 5′-AAAGGATGCGGTGTCATTAT-3′ (reverse). The PCR assay was run on a 1% agarose gel from which a PCR product of approximately 1300 bp was sliced and extracted (Qiagen, The Netherlands) and subsequently cloned into the pCR-II-Blunt TOPO vector (Invitrogen, Inc, CA). The zebrafish *dyx1c1* clone was then validated using Sanger sequencing (Eurofin MWG Operon).

### Quantitative Real Time PCR (qPCR)

Tissues including eyes, brain, heart, gill, swimbladder, liver, kidney, ovary, and testis were isolated from adult (1 year old, AB strain) male and female zebrafish and pooled by tissue type for RNA extraction. Embryos of different stages of development including <3 and 10 hpf and 1–5 dpf were collected for RNA extraction. RNA extraction, cDNA synthesis and real-time PCR assays were performed as described previously [33]. For measuring the transcript levels of *dyx1c1*, F- 5′-CACCGCGCTCAGAGAGTC-3′ and R-5′TCCGCCTGCTTCTTCAAC-3′ were used as forward and reverse primers respectively. β-actin was used as the internal reference control to normalize the expression levels of *dyx1c1*. RNA from the stage before zygotic transcription (<3 hpf) and eye were used as calibrators in qPCR analysis in embryos and adult tissues. Each PCR assay was performed on two independent sets of cDNA samples in triplicates.

### Whole-mount in situ Hybridization

Whole-mount in situ hybridization was carried out using digoxigenin-labeled riboprobes as previously described [34]. Antisense RNA probe for zebrafish *dyx1c1* was in vitro synthesized by T7 polymerase after linearising the template with SpeI. Riboprobes for the laterality markers, *otx5* [35], *foxa3* [36] and *lov* [37] were generated as described previously. Images of the stained embryos were obtained using a Nikon digital camera.

### Morpholino Based Knockdown and Rescue Experiments

Morpholinos targeting the ATG translation start site (ATGMO; 5′- GTGATCTCTCACTATCAGCGGCATC-3′), exon 2-intron 2 splice site (SPMO; 5′-TGACAGTCAACATGTCTTACCGATG-3′), a 5 bp mismatch control MOs (misATGMO; 5′-GTCATGTCTCAGTATCACCCGCATC-3′, misSPMO; 5′- TGACACTGAACATCTCTTAGCCATG-3′), a standard control MO (5′-CCTCTTACCTCAGTTACAATTTATA-3′) and a p53MO (5′-GCGCCATTGCTTTGCAAGAATTG-3′) were purchased from Genetools, (Genetools Inc, OR, USA). MOs dissolved in 1× Danieau buffer were injected into zebrafish embryos at 1–2 cell stages at varying concentrations of 50, 100, 150, 200 and 250 µM. To test the off-target effects of MOs, 200 µM concentration of p53MO was injected alone or together with 100 µM each of ATGMO+SPMO. To confirm the specificity of the *dyx1c1* MOs (ATGMO and SPMO), a 5′ capped mRNA of *dyx1c1* was synthesized using mMessage Machine kit (Ambion, TX, USA) and coinjected along with the *dyx1c1* MOs. Splice morpholino specificity was confirmed by RT-PCR using primers spanning exon 2 (F- 5′-GAGATGAAATGTGGGAGCAG-3′) and exon 5 (R-5′-ACTCGTGGAGTGAAGCTGAT-3′) of *dyx1c1* gene. PCR was carried out using the amplification program that consists of 4 min of initial denaturation at 95°C, 40 cycles of 30 s at 95°C, 30 s at 57°C for *dyx1c1* and 58°C for *β-actin*, 60 s at 72°C followed by 10 min of final extension at 72°C.

### Acridine Orange Staining

Apoptotic cell death was detected by acridine orange staining following the protocol described previously [38]. All fluorescent images were accquired with Leica camera mounted on a Leica stereomicroscope.

### Histology

Zebrafish embryos (2 dpf and 3 dpf) were fixed in 4%PFA overnight at 4°C. The fixed specimens were then serially dehydrated in 70%, 80%, 95% and 100% ethanol for 5 min each, treated with xylene twice for 10 min and embedded in paraffin. Paraffin blocks were then sectioned using a microtome at 3 µm thickness and the sections were stained with Hematoxylin and Eosin.

### Immunohistochemistry

Embryos were fixed in 4% PFA overnight at 4°C. Fixed embryos were rinsed four times in PBS and permeabilized in cold acetone for 20 min at −20°C. Embryos were then washed in PBS and blocked for 2 h at RT in blocking solution (1× PBS, pH 7.4, 1% DMSO, 0.5% Tween20, 1% BSA, 10% normal goat serum). Following blocking, embryos were incubated with anti-acetylated tubulin antibody (1∶1000, Sigma-Aldrich, St Lois, MO) at 4°C overnight. Goat anti-mouse Alexa 488 was used as the secondary antibody (1∶500, Invitrogen, Inc, CA, USA). For nuclear staining of KV cells, 14 hpf embryos were first immunostained with anti-acetylated tubulin antibody as mentioned above and subsequently stained with DAPI (5 mg/ml, Molecular Probes) at 1∶50 dilution in dark for 10 min at RT. The confocal images were obtained with Andor spinning disk confocal microscope using a 60× water objective.

### Transmission Electron Microscopy

Three days old embryos were fixed in 2% glutaraldehyde solution containing 1% PFA in 0.1 M phosphate buffer (PB), pH 7.4 at RT and stored at 4°C until use. Specimens were rinsed in 0.1M PB, pH 7.4 and then post-fixed with 2% osmium tetroxide in 0.1M PB, pH 7.4 at 4°C for 2 h, dehydrated in ethanol followed by acetone and then embedded in LX-112 (Ladd, Burlington, Vermont, USA). Semithin sections were cut and stained with toluidine blue O and used for light microscopic analysis. Ultrathin sections of approximately 40–50 nm were cut by a Leica EM UC 6 (Leica, Wien, Austria) and stained with uranyl acetate followed by lead citrate and examined in a Tecnai 10 transmission electron microscope (FEI Company, Eindhoven, The Netherlands) at 100 kV. Images were acquired using a Veleta camera (Olympus Soft Imaging Solutions, GmbH, Münster, Germany).

## Results

### Identification, Cloning and Sequence Analysis of *dyx1c1* Gene in Zebrafish

**Figure 1:**
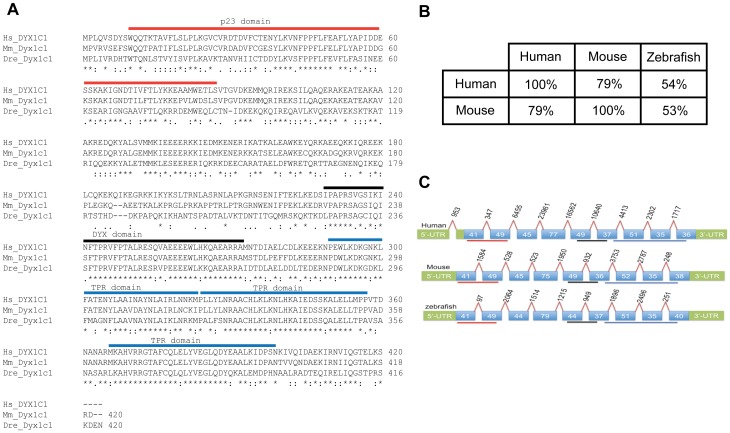
Sequence alignment and genomic structure of the vertebrate *DYX1C1*/*Dyx1c1*/*dyx1c1* genes and proteins. Sequence comparison of zebrafish (*Danio rerio*; Dre) Dyx1c1 protein with that of Human (*Homo sapiens*; Hs) and mouse (*Mus musculus*; Ms) protein sequences (A). The p23, DYX and TPR domains are denoted by red, black and blue lines respectively. Protein sequence identity shown in percentage for the three species (B). The comparison of *dyx1c1* cDNAs from Human, mouse and zebrafish reveals 9 exons intercepted by 8 introns (C). Human cDNA sequence shows the presence of an additional intron in the 5′untranslated region (UTR). The UTRs are denoted by green boxes, exons by blue boxes with the number of amino acids they code and introns are represented by slanting lines connecting the exons with the number of nucleotides (nt) on top. The conserved domains are denoted by red (for p23 domain), black (for DYX domain) and blue (for TPR domain) lines below the cDNA structure for each species.

By searching the zebrafish genome database, we identified the zebrafish ortholog of *dyx1c1* (ENSDARG00000007792). The zebrafish *dyx1c1* is located on chromosome 13 and is predicted to encode a protein of 420 amino acids. Comparison of the zebrafish Dyx1c1 protein sequence with that of human and mouse revealed 54% and 53% sequence identity, respectively (Fig. 1A, B). In addition, we found a high degree of conservation in the intron-exon architecture of zebrafish *dyx1c1* with its mammalian counterparts (Fig. 1C). The protein coding region comprised 9 exons separated by 8 introns. Although the length of the introns varied between the three species, their positions were conserved. The exons also displayed high similarity in the number of amino acids they coded for; exons 1, 2 and 8 coded for the same number of amino acids in all the species, whereas exons 3 and 5 coded for the same number of amino acids in human and mouse but not in zebrafish. The total number of amino acids coded by exons 6 and 7 were the same in human and zebrafish but not in mouse. Exon 4 and 9 of the protein coding region showed the most variability among the three species analyzed. The DYX and TPR domains were highly conserved between the three species, whereas the zebrafish p23 domain was less conserved with respect to the mamalian p23 domain. However, the three domains spanned across the same exons in all the species (Fig. 1C). Analyses of the genomic region near *dyx1c1* revealed synteny between zebrafish and humans both including genes coding for ccpg1, pygo1 and prtg (data not shown).

Using qPCR, the coding region of *dyx1c1* was amplified from embryos at 50% epiboly stage. The amplified fragment was then purified and cloned into a TOPO vector.

### Expression of *dyx1c1* in Developing Embryos and Adult Organs

**Figure 2:**
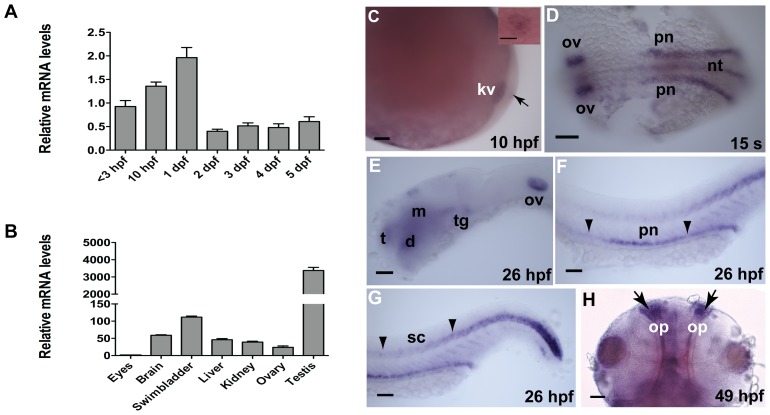
Expression of *dyx1c1* mRNA during embryonic development and in adult tissues. qPCR analysis of the transcript levels of *dyx1c1* during embryonic development (A) and in adult tissues (B). Whole-mount *in situ* hybridization showed that *dyx1c1* is expressed in KV at 10 hpf (C). Inset in C is a close-up view of KV. At 15-somites, *dyx1c1* was expressed specifically in the otic vesicle, pronephros and neural tube (D). At 26 hpf *dyx1c1* was detected in the brain and is still maintained in otic vesicle, pronephros and spinal canal (E–G). Later at 49 hpf, *dyx1c1* was visible in the olfactory placode (H). Panels E–G show lateral views of embryos. Panels D and H show dorsal and ventral views of embryos, respectively. Scale bars indicate 100 µm. Abbreviations: KV, Kupffer’s vesicle: nt, neural tube: pn, pronephros: t, telencephalon: d, diencephalon: m, midbrain: tg, tegmentum: ov, otic vesicle: sc, spinal canal: op, olfactory placode.

Using qPCR, we measured the mRNA levels of *dyx1c1* in developing embryos and in early larval stages. Zebrafish *dyx1c1* was maternally expressed as seen at <3 hpf, a stage before mid-blastula transition (MBT) and its expression levels increased until 1 dpf. The mRNA levels of *dyx1c1* dropped down by 2 dpf and remained at low levels during larval stages (Fig. 2A). To further analyze the spatial expression of *dyx1c1*, we performed whole-mount *in situ* hybridization on developing embryos. At 10 hpf, a weak expression of *dyx1c1* was detected in KV (Fig. 2C). At 15 somites stage, *dyx1c1* expression was localized in the otic vesicle, pronephros and neural tube (Fig. 2D). By 26 hpf, *dyx1c1* expression was evident in several regions of the brain including telencephalon, midbrain, and tegmentum and was persistently expressed in the otic vesicle, spinal canal and pronephros (Fig. 2E–G). However the expression of *dyx1c1* was reduced by 49 hpf and was detected strongly only in the olfactory placode (Fig. 2H). It is evident from RNA *in situ* hybridization that *dyx1c1* is expressed in ciliated tissues suggesting that it might have a role in cilia development and/or function.

The relative mRNA levels of *dyx1c1* in different pooled tissues of adult male and female zebrafish were measured by qPCR. Highest expression of *dyx1c1* was seen in the testis. *dyx1c1* was also detected in other organs including brain, swimbladder, liver, kidney and ovary (Fig. 2B). Our results are consistent with the expression analysis of *DYX1C1* in human tissues [1].

### Knockdown of *dyx1c1* Leads to Body Curvature, Hydrocephalus and Kidney Cysts

**Figure 3:**
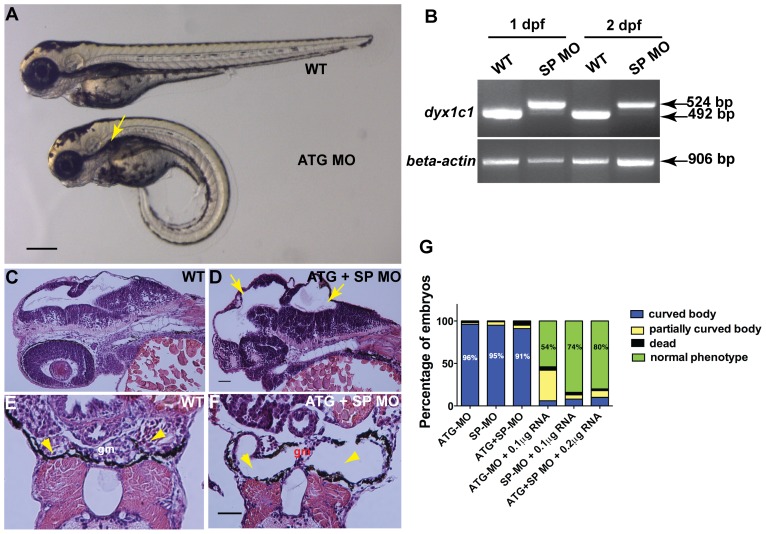
Knockdown of *dyx1c1* showed typical cilia phenotypes. Injection of ATGMO or SPMO at 200 µM concentration produced ventrally curved body axis, hydrocephalus and kidney cysts (A). Arrow in panel A denotes kidney cyst in ATG morphant. RT-PCR showed aberrant splice transcripts in SPMO injected embryos at 1 and 2 dpf (B). Histological sections of 2 day old embryos injected with both ATGMO and SPMO (100 µM each) showed hydrocephalus (D; yellow arrows) compared to normal size brain ventricles in WT (C). Transverse histological sections across the pronephros at 3.5 dpf showed normal pronephros in WT embryos (E). Section of *dyx1c1* morphant (ATGMO+SPMO) showed severe pronephric distention and a thin glomerulus in the center (F). Yellow arrowheads in panel E and F point out normal pronephros in WT and dilated pronephros in morphant embryo, respectively. Quantitative analysis of the rescue of *dyx1c1* morphant phenotype to WT phenotype with different combinations of MOs and *dyx1c1* mRNA (G). Scale bars indicate 100 µm. Abbreviation: gm, glomerulus.

To investigate the biological role of *dyx1c1* during embryonic development in zebrafish we used MOs to knockdown the protein expression of Dyx1c1. Both translation start-site MO (ATGMO) and splice-site MO (SPMO) targeting the splice junction of exon 2 and intron 2, were injected alone into 1-cell embryos at varying concentrations of 50, 100, 150, 200 and 250 µM. The efficiency of the MOs was dose dependent; at 50 µM dose no visible morphological defects were observed whereas at higher concentrations, both the MOs resulted in similar phenotypes as described below (Fig. 3A, S1). At the highest concentration, the MOs increased the percentage of mortality in the morphants to more than 70%.

The effects of the loss-of-function of *dyx1c1* were observed in higher percentage (>90%) of morphants at 200 µM for both the MOs. The knockdown efficiency of SPMO was strong at 1 dpf but decreased at 2 dpf. As both ATGMO and SPMO resulted in identical phenotypes we used a combined dose of 100 µM each to get a total of 200 µM for all experiments unless otherwise specified. For all experiments, a total concentration of about 8 ng of ATGMO or SPMO or combination of both (4 ng each) was injected per embryo. The 5 bp mismatch control MO (misATGMO and misSPMO) that were tested at the same concentration range had no effects at 50 and 100 µM but produced a phenotype identical to that seen with ATGMO or SPMO in 10% of the injected embryos at 200 µM. Microjection of a standard control MO (cMO) had no effects at the same concentration range and the injected embryos appeared normal like the wild-type uninjected embryos. Wild-type (WT) embryos were used as controls in all experiments.

The specificity of the SPMO was confirmed by RT-PCR which revealed aberrant splicing events in 1 and 2 dpf morphants. Sequencing of the misspliced transcript revealed the inclusion of 32 bp from intron 2 which might encode nonfunctional truncated Dyx1c1 protein in the splice morphants (Fig. 3B). However at 3.5 dpf the efficiency of SPMO was reduced in embryos showing weak phenotype (Fig. S1). We therefore used only morphants showing prominent body curvature, hydrocephalus and kidney cysts for all our experiments. Coinjection of 5′capped mRNA of zebrafish *dyx1c1* along with 200 µM of ATGMO or SPMO or combination of both, rescued the curved body axis phenotype to normal WT phenotype (Fig. 3G).

Wild-type embryos injected with ATGMO and SPMO either alone or together developed numerous morphological changes that were visible from 1 dpf onward. *dyx1c1* morphants developed ventrally curved body axis, hydrocephalus and pronephric cysts which became visible only at 3 dpf (Fig. 3A). Additionally, upon mechanical stimulus the morphants exhibited abnormal circular swimming behaviour. Histological analysis of transverse sections of WT and *dyx1c1* morphant clearly showed hydrocephalus in 2 days old morphant embryos and dilated pronephric tubules in 3.5 dpf morphant embryos (Fig. 3C–F).

One of the major off-target effects caused by MOs is p53 mediated cell death [38]. Thus, to further confirm that the MOs are specifically targeting *dyx1c1,* we performed acridine orange staining on wild-type, *p53* morphants, *dyx1c1* morphants and p53MO coinjected embryos to visualize apoptosis. Our results show that coinjection with p53MO does not produce any phenotypic changes in *dyx1c1* morphants, thus confirming that the phenotypes are caused by true loss-of-function of *dyx1c1* (Fig. S2).

### Loss-of-function of *dyx1c1* in Zebrafish Leads to *situs inversus* in Different Organs

**Figure 4:**
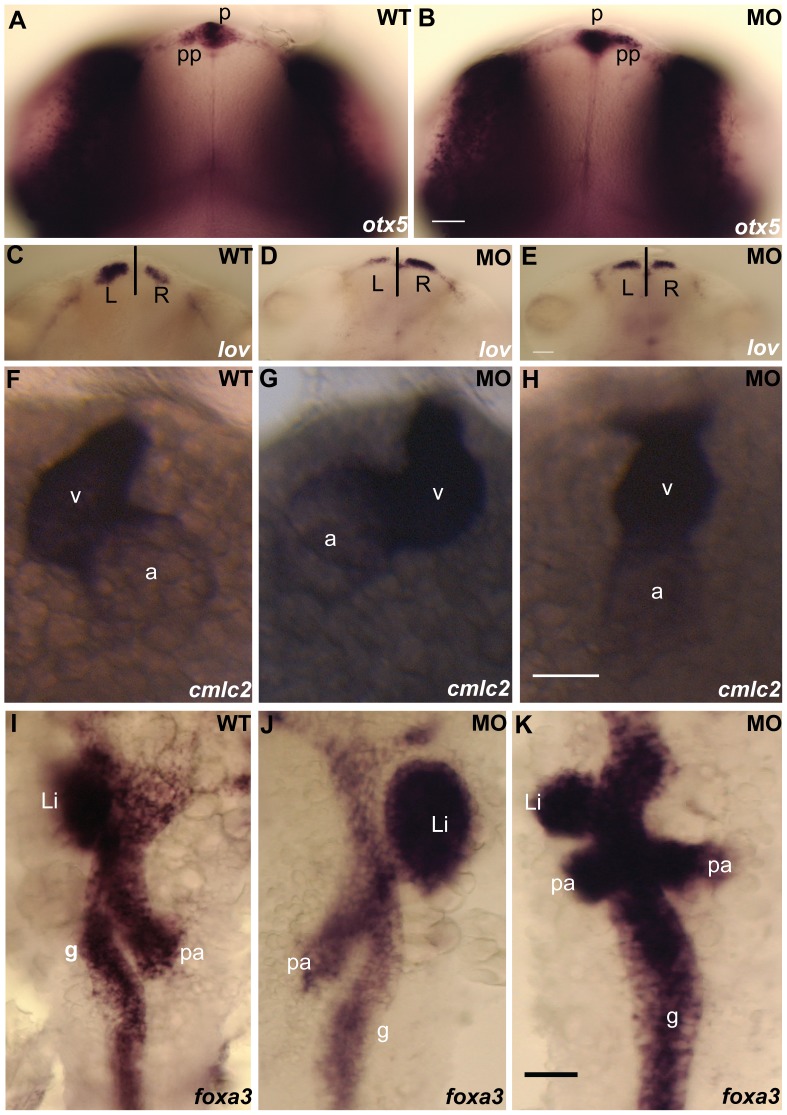
Loss-of-function of *dyx1c1* caused L–R assymetry defects in zebrafish. Whole-mount in situ hybridization showing the expression of laterality markers in WT and *dyx1c1* morphants (ATGMO+SPMO) at 2 dpf. The position of the asymmetrically placed organs was irregular in at least 50% of the embryos injected with *dyx1c1* MO. *otx5* expression showing the left sided placement of the parapineal organ in WT (A). In *dyx1c1* morphant, the parapineal position was reversed (B). *lov* was strongly expressed in the left habenular in WT (C). In *dyx1c1* morphant *lov* expression was stronger in the right or expressed symmetrically (D & E). Knockdown of *dyx1c1* altered heart looping. Ventral views of embryos at 2 dpf (F–H). *cmlc2* expression showed normal heart looping in WT (F). Cardiac looping was reversed (G) or absent in *dyx1c1* morphant embryos (H). Expression of *foxa3* revealing the position of the gut, liver and pancreas in WT embryo (I). The positions of the liver, gut and pancreas were irregular in *dyx1c1* morphants (J & K). Panels A, B, C, D, E, I, J and K are dorsal views with anterior to the top. Frontal views are shown in panels F–G. Scale bars indicate 50 µm. Abbreviations: p, pineal organ: pp, parapineal organ: v, ventricle: a, atrium: li, liver: pa, pancreas: g, gut.

Zebrafish cilia mutants display phenotypes including curvature of the body axis, hydrocephalus and kidney cysts similar to that of *dyx1c1* morphant embryos suggesting a potential role for *dyx1c1* in cilia formation and/or function [31]. As cilia have been implicated in the specification of left-right (LR) asymmetry of the body plan, we sought to analyze the left-right patterning in asymmetrically placed organs such as the heart, epithalamus and visceral organs. The percentage of morphant embryos showing randomization in LR asymmetry in different organs is shown in Table 1. To assess brain asymmetry, the expression of markers for parapineal (*otx5*) and habenular nuclei (*lov*) were analyzed by *in situ* hybridization on two days old embryos [35], [37]. In WT control embryos, the normal position of the parapineal organ is on the left side, whereas in 41% of the *dyx1c1* morphant embryos it was reversed (Fig. 4A, B). The expression of *lov* was stronger on the left habenula in WT control embryos but 42% of the *dyx1c1* morphants showed stronger expression of *lov* on the right habenula and in 5% of the morphants *lov* was symmetrically expressed (Fig. 4C–E). Using *cardiac myosin light chain 2* (*cmlc2)* gene expression as marker, we determined the heart looping in WT and *dyx1c1* morphant embryos [39]. Normal heart looping, with left placement of the ventricle and right placement of the atrium was observed in 98% of wild-type embryos, whereas, in 26% of the *dyx1c*1 morphants the looping was inverted and in 24% of morphant embryos the heart failed looping at all (Fig. 4F–H). Assessment of the positioning of the visceral organs in WT embryos and morphants using the endodermal marker *foxa3* revealed that the position of the gut, liver and pancreas was irregular in the morphants [36]. About 52% of the *dyx1c1* morphants showed situs inversus and 5% showed heterotaxia with respect to the positioning of the liver (Fig. 4I–K).

**Table 1:** Percentage of embryos showing normal or altered organ laterality in WT and *dyx1c1* morphants.

Markers		Normal	Reversed	Bilateral	Absent
*otx5*	WT (n = 94)	97%	3%	–	–
	**MO (n = 120)**	**58%**	**41%**	–	–
*lov*	WT (n = 81)	98%	2%	–	–
	**MO (n = 81)**	**53%**	**42%**	**5%**	–
*cmlc2*	WT (n = 131)	97%	3%	–	–
	**MO (n = 152)**	**48%**	**26%**	–	**24%**
*foxa3*	WT (n = 87)	98%	2%	1%	–
	**MO (n = 101)**	**47%**	**52%**	**5%**	–

Asymmetry of the parapineal organ and habenular nuclei was detected by *otx5* and *lov* expression. Bilateral expression of *lov* in morphants refers to equal expression of *lov* on both left and right habenula. Heart looping in WT and morphant was detected using *cmcl2*. About 24% of morphant embryos showed no looping which is denoted as absent in the table. Randomization of the liver position is quantified with *foxa3* expression.

### Loss-of-function of *dyx1c1* Affects Cilia Length in Multiple Organs

**Figure 5:**
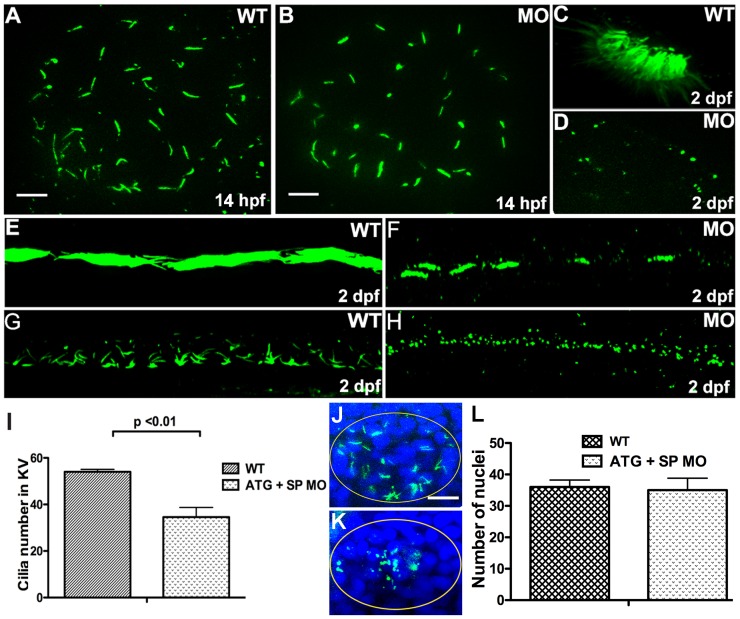
Knockdown of *dyx1c1* reduced cilia length and number in different organs Immunolabelling of KV cilia with anti-acetylated tubulin in WT (A) and *dyx1c1* morphants (B) at 14 hpf showed reduction in cilia length in morphant embryos. Compared to WT control (C), morphant embryo (D) revealed fewer and shortened cilia in the olfactory placode. At 2 dpf, cilia in *dyx1c1* morphants were shortened in pronephros and spinal canal (F, H) compared to WT (E, G). Measurement of cilia number in WT and morphants (I). Confocal images of KV in WT and morphant embryos at 14 hpf stained with anti-acetylated tubulin and DAPI (J, K). Graphical representation of total number of nuclei/KV in WT and morphant embryos (L). Panel C, D, E, F, G and H are lateral views with anterior to the left in E, F, G and H. Scale bars indicate 10 µm.

The KV in zebrafish is a transient ciliated organ which is essential for specifying organ laterality [40]. To test whether the L–R asymmetry defects observed in *dyx1c1* morphants were due to impaired ciliogenesis, we performed immunolabelling with an antibody against acetylated tubulin to visualize cilia in KV. Measurement of cilia number in KV of 10-somite stage WT and morphant embryos revealed that cilia number was significantly reduced in *dyx1c1* morphants when compared to those of the WT embryos (Fig. 5A, B, I). Examination of DAPI stained KV cell nuclei showed no difference in the total number of cells in KV between WT and morphant embryos suggesting that reduction in cilia number in morphant embryos is not produced by loss of ciliated cells (Fig. 5J–L). We also found slight reduction in cilia length in KV of *dyx1c1* morphants (Fig. 5A, B).

The expression of *dyx1c1* in many ciliated organs such as the pronephros, spinal canal, otic vesicle and olfactory placode prompted us to examine cilia in these organs. In zebrafish, cilia in the pronephros and spinal canal are motile and are essential for driving fluid flow within these organs. Disruption of cilia structure or motility in the pronephros and spinal canal leads to fluid accumulation and organ distention subsequently resulting in kidney cysts and hydrocephalus respectively [41]. As expected the proximal region of WT zebrafish pronephros was densely populated with cilia that were long and extended into the lumen of the kidney tubule. Interestingly, the pronephric cilia of *dyx1c1* morphants were less dense and were severely shortened in length (Fig. 5E, F). Likewise, spinal canal cilia of *dyx1c1* morphants were dramatically reduced in length in contrast to that seen in WT embryos (Fig. 5G, H). We next visualized cilia in the olfactory placode of 2 dpf WT and morphant embryos. We found in the WT embryos numerous cilia projecting from the epithelial cells of the olfactory placode. Strikingly, we detected very few olfactory cilia in the *dyx1c1* morphants and those present were severely shortened in length (Fig. 5C, D). Together, these data suggest that *dyx1c1* might have a central role in regulating cilia length in many organs in zebrafish.

### Zebrafish *dyx1c1* is Needed for Dynein Arm Assembly

**Figure 6:**
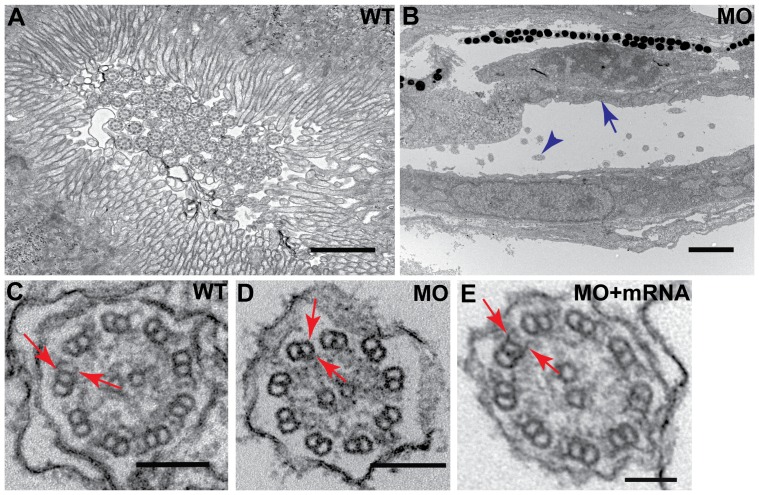
Disruption of *dyx1c1* leads to pronephric brush border defects and loss of cilia dynein arms. Ultrastructure of WT (3.5 dpf) pronephric duct showed dense brush border of apical microvilli extending from the epithelial cells and the presence of numerous cilia in the lumen (A). Apical microvilli were absent in *dyx1c1* morphants (ATGMO+SPMO) at 3.5 dpf (B). Arrow in blue denotes missing brush border and blue arrowhead denotes cilia. Electron micrography of pronephric cilia axoneme in *dyx1c1* morphant embryo at 3.5 dpf showed the absence of both ODA and IDA on the outer microtubules (D) whereas the dynein arms were present in WT embryos (C). Coinjection of *dyx1c1* mRNA with MO rescued both ODA and IDA (E). Arrows in red indicate ODA and IDA in WT, morphant and rescued embryo. Scale bars in panels A and B indicate 1 µm and 2 µm respectively. Scale bars in panels C and D are 100 nm and 200 nm in E.

Transmission electron microscopy was used to examine the ultrastructural defects in cilia to better understand the functional role of *dyx1c1* in ciliogenesis. The cilium consists of 9 microtubule doublets (A and B subunits) arranged in the border and two central tubules. Each outer A subunit has a set of arms attached, IDA and ODA. Lack of dynein arms have been shown to render cilia immobile [42]. We analyzed ultrathin sections of the pronephros because zebrafish pronephros is a multiciliated tissue providing easy access to examine motile cilia, and secondly, because kidney cysts, a manifestation of defective cilia motility, were apparent in *dyx1c1* morphants from 3 dpf onward. In WT embryos staged at 3.5 dpf, a dense brush border of apical microvilli projecting from the epithelial cells and numerous cilia were evident in the pronephric duct (Fig. 6A). However in the morphant embryos the pronephric duct was dilated and the epithelia completely lacked microvilli (Fig. 6B). Furthermore, we observed abnormal cilia axoneme structure in the morphants. In most of the *dyx1c1* morphants, both ODA and IDA were absent as compared to WT controls (Fig. 6C, D). Ocassionally, in some morphants remnants of ODA were noticeable but the IDA were still missing (data not shown). As in the pronephros, dynein arms were also missing in the olfactory placode cilia (Fig. S3).

### *dyx1c1* mRNA Rescues Cilia Defects in *dyx1c1* Morphants

To confirm that the cilia defects observed were due to true loss-of-function of *dyx1c1*, we performed rescue experiments by coinjecting *dyx1c1* mRNA with ATGMO+SPMO. The laterality defects caused by suppression of *dyx1c1* was greatly reduced by *dyx1c1* mRNA injection (Fig. S4). Also, the dramatic reduction of cilia length in the pronephros was rescued by coinjection of *dyx1c1* mRNA (Fig. S4). Ultrastructural analyses revealed that the loss of dynein arms caused by knockdown of *dyx1c1* was rescued efficiently in the pronephric cilia as well as in the olfactory placode cilia by coinjection of *dyx1c1* mRNA with ATGMO+SPMO (Fig. 6E, Fig. S3).

## Discussion

In the present study, we show that the ortholog of the dyslexia candidate gene *dyx1c1* is essential for cilia growth and motility in zebrafish. Cilia are membrane-bound organelles that are crucial for vertebrate development, organ morphogenesis and differentiation of sensory cells. Ciliary dysfunction has been described in a dozen of human ciliopathies such as primary ciliary dyskinesia (PCD), Autosomal Dominant Polycystic Kidney Disease (ADPKD), Nephronophthisis (NPHP), Bardet Biedl Syndrome (BBS) and Jouberts syndrome (JBTS) and the list continues to expand as more ciliary proteins are being identified [43], [44], [45], [46]. Zebrafish has been used extensively to study cilia structure, function and signalling and several cilia mutants have been generated. Interestingly, the ciliary mutants and morphants with defects in diverse ciliary genes display defects including body curvature, hydrocephalus, kidney cysts and left-right asymmetry [47], [48], [49], [50]. Here, we demonstrate that zebrafish *dyx1c1* is specifically expressed in ciliated tissues and its inhibition results in ciliopathy-related phenotypes consistent with those seen in zebrafish cilia mutants [31], [49], [51].

Beating of the motile ependymal cilia in the brain propels cerebrospinal fluid (CSF) flow in brain ventricles and impairment of ependymal cilia has been linked to the formation of hydrocephalus in different animal models [52], [53], [54]. In humans, hydrocephalus has been noticed in some patients with PCD [55]. The presence of renal cysts in patients of cystic kidney diseases has been suggested to result from the functional failure of renal primary cilia, possibly through abnormal cilia-mediated Ca^2+^ signalling as suggested by *in vitro* studies [56], [57]. Spinal cord cilia and renal cilia in zebrafish are motile and their beating is essential to move fluid within these organs [41]. Our observation of hydrocephalus and pronephric cysts in *dyx1c1* morphants strongly suggested that cilia motility might be compromised in these organs.

We report randomization of LR asymmetry of different organs in *dyx1c1* morphant embryos (Fig. 4) and cilia in many organs including the spinal canal, pronephros and olfactory placode appeared greatly stunted (Fig. 5). Significant reduction in cilia number was also detected in the KV and olfactory placode. It is important to note that among all tissues examined, KV cilia was least affected which could possibly be due to the incomplete suppression of maternally supplied mRNA not affected by SPMO. Interestingly, laterality defects have been observed in PCD patients and also in mice carrying mutation in genes implicated in cilia function [58], [59]. The unique motile cilia of zebrafish KV, an organ homologous to mouse node function to set up left-right asymmetry of organs in zebrafish [40]. We suggest that the fewer number of short cilia in KV of *dyx1c1* morphants may not be able to generate a clockwise fluid flow within this organ, which is required for specifying LR organ asymmetry. Supporting our hypothesis, cilia length has been shown previously to play a crucial role in regulating speed and direction of fluid flow inside KV [60]. Previous work has shown that zebrafish mutants showing ciliary length defects also exhibit kidney cysts and hydrocephalus [31], [48], [49], [51]. Taken together, these results suggest that *dyx1c1* could be important for controlling cilia length in different organs in zebrafish.

Structural abnormalties of cilia have been previously shown to affect cilia motility [61], [62]. Human PCD subjects with mutations in the *DNAAF3* gene lack ODA and IDA in their cilia, a phenotype which could also be reproduced in zebrafish by morpholino knockdown of *dnaaf3* [63]. Thus, absence of dynein arms in *dyx1c1* morphants suggests that *dyx1c1* might be required for cilia movement through maintenance of the assembly of ODA and IDA. Interestingly, some zebrafish mutants and morphants that lack both ODA and IDA do not have short cilia whereas mutations in ciliary genes in *chlamydomonas* that show defects in outer or inner dynein arms do cause a reduction in the average length of the flagella [63], [64], [65], [66]. Elongation and maintenance of cilia axoneme is dependent on the bidirectional intraflagellar transport (IFT) of IFT protein complex. Chlamydomonas ODA16 is an IFT associated protein that is needed for the proper assembly of outer dynein arm proteins. Short cilia observed in *dyx1c1* morphants could possibly result from the disruption of IFT and perhaps *dyx1c1* functions in zebrafish to transport specific components of outer and inner dynein arms to the ciliary axoneme [66], [67]. Molecular chaperones such as HSP90, HSP70 and TCP1 are known to modulate the assembly of cytoskeletal proteins [68]. Similar to other family of TPR domain containing proteins, DYX1C1 has been shown to act as a co-chaperone for HSP70 and HSP90 [69]. Interestingly, the DYX1C1 protein interactome in SH-SY5Y cells by Tammimies et al., reveals that DYX1C1 interacts with several microtubule and cytoskeletal proteins [70]. Based on the accumulated evidence and our *in vivo* data, we hypothesize that DYX1C1 might function to deliver structural and functional components of the axoneme from the cytoplasm to the axoneme by interacting with proteins essential for IFT. Further studies are needed to test this hypothesis.

A role for cilia in dyslexia has not been shown until recently. DCDC2 was the first dyslexia protein shown to regulate ciliary signalling [24]. A recent report using a large-scale gene coexpression analysis of microarray data sets from human ciliated tissues suggest that *DYX1C1, KIAA0319* and *DCDC2* have a role in ciliary functions [71]. Also, an earlier comparative genomic screen identified DYX1C1 as one of the proteins involved in ciliary and basal body biogenesis [72]. A recent study on the transcriptional profiling of multiciliated mouse tracheal epithelial cells showed that Dyx1c1 is localized to centrosome and cilia and upregulated during ciliogenesis [73]. Our results in zebrafish provide direct evidence for the role of the *dyx1c1* in the biology and function of cilia suggesting a similar function for DYX1C1. Disruption of *DYX1C1*, *DCDC2* and *KIAA0319* individually results in neuronal migration abnormalities in rat neocortex. Whether the neuronal phenotype observed in animal models and in human dyslectic brains is a consequence of defects in neuronal cilia structure and function remains unknown. However, the significance of primary cilium in brain patterning including cortical morphogenesis has been implicated previously [28], [74]. Cognitive impairments are often observed in ciliopathy syndromes such as Joubert Syndrome, Bardet-Biedl Syndrome and Alström Syndrome and it is possible that these symptoms are manifestations of defects in neuronal cilia [75]. Until now it was not clear whether migrating neurons possess cilia, but a recent study in mouse suggests that cilia growth occurs only after the completion of neuronal migration in the cortex [76]. Thus, the function of primary cilia in migration of neurons still remains poorly characterized and further studies with respect to cilia biology in the CNS are required.

Centrosome positioning is considered as a crucial factor for proper migration of neurons in the cortex. In our previous work we have shown that DYX1C1 localizes to the centrosome and also interacts with Lissencephaly 1 (LIS1), another centrosomal protein. Mutations in LIS1 lead to a severe neuronal migration disorder lissencephaly in humans [70], [77]. However, it remains to be determined if this interaction regulates centrosomal orientation during the migration of neurons.

The zebrafish is a very powerful model to study ciliopathies. In this work we show for the first time the involvement of *dyx1c1* in ciliary function in an animal model. With direct evidence implicating now DYX1C1 and DCDC2 in ciliary functions and the bioinformatic suggestion of a role for KIAA0319 as well, we propose that dyslexia should become considered as a new type of ciliopathy.

## Supporting Information

Figure S1 **Morphological phenotypes induced by ATGMO and SPMO.** Both ATGMO and SPMO when injected alone produced identical phenotypes. Hydrocephalus and kidney cysts were clearly visible in the morphants at 2 dpf (B & C) and 3 dpf (E & F) respectively as compared to the nomal phenotype in wild-type (A & D). Arrows denote hydrocephalus and kidney cysts are denoted by arrowheads. RT-PCR showing the efficiency of SPMO at 3.5 dpf in embryos showing strong and weak phenotypes (G). Scale bars indicate 100 µm.
(TIF)

Figure S2 **Morpholino specificity confirmed by coinjection with p53.** Analysis of apoptotic cell death in WT (A–D), p53 morphants (E–H), *dyx1c1* morphants (ATGMO+SPMO; I–L) and *dyx1c1*+ *p53* morphants (M–P). Bright field (A,E,I,M) and fluorescent images of wild-type and morphants (B–D, F–H, J–L, N–P) at 1 dpf. Fluorescent signal in *dyx1c1* morphants appeared similar to that seen in WT. *dyx1c1* morphant phenotype was not affected by p53 coknockdown. Scale bars indicate 100 µm.
(TIF)

Figure S3 **Dynein arms of olfactory cilia affected in** ***dyx1c1*** **morphants.** Ultrastructure of olfactory cilia at 3 dpf showed loss of ODA and IDA in *dyx1c1* morphants (B) as compared to WT (A). Coinjection with *dyx1c1* mRNA rescued both the dynein arms (C). Arrows denote ODA and IDA. Scale bars indicate 200 nm.
(TIF)

Figure S4 ***dyx1c1*** **mRNA rescues cilia defects in** ***dyx1c1*** **morphants.** Anti-acetylated tubulin staining of cilia in the pronephros of wild-type (A), *dyx1c1* morphant (B) and mRNA coinjected embryo (C). Percentage of embryos showing left-side (normal), right-side (situs inversus) placement of liver and heterotaxia in wild-type, *dyx1c1* morphants and mRNA injected embryos (D). Scale bar indicate 10 µm.
(TIF)
