# Supplementary figures and images for: The Zebrafish Orthologue of the Dyslexia Candidate Gene DYX1C1 Is Essential for Cilia Growth and Function
Source: PLoS One. 2013 May 1;8(5):e63123. doi: 10.1371/journal.pone.0063123 (PMC3641089; doi:10.1371/journal.pone.0063123)

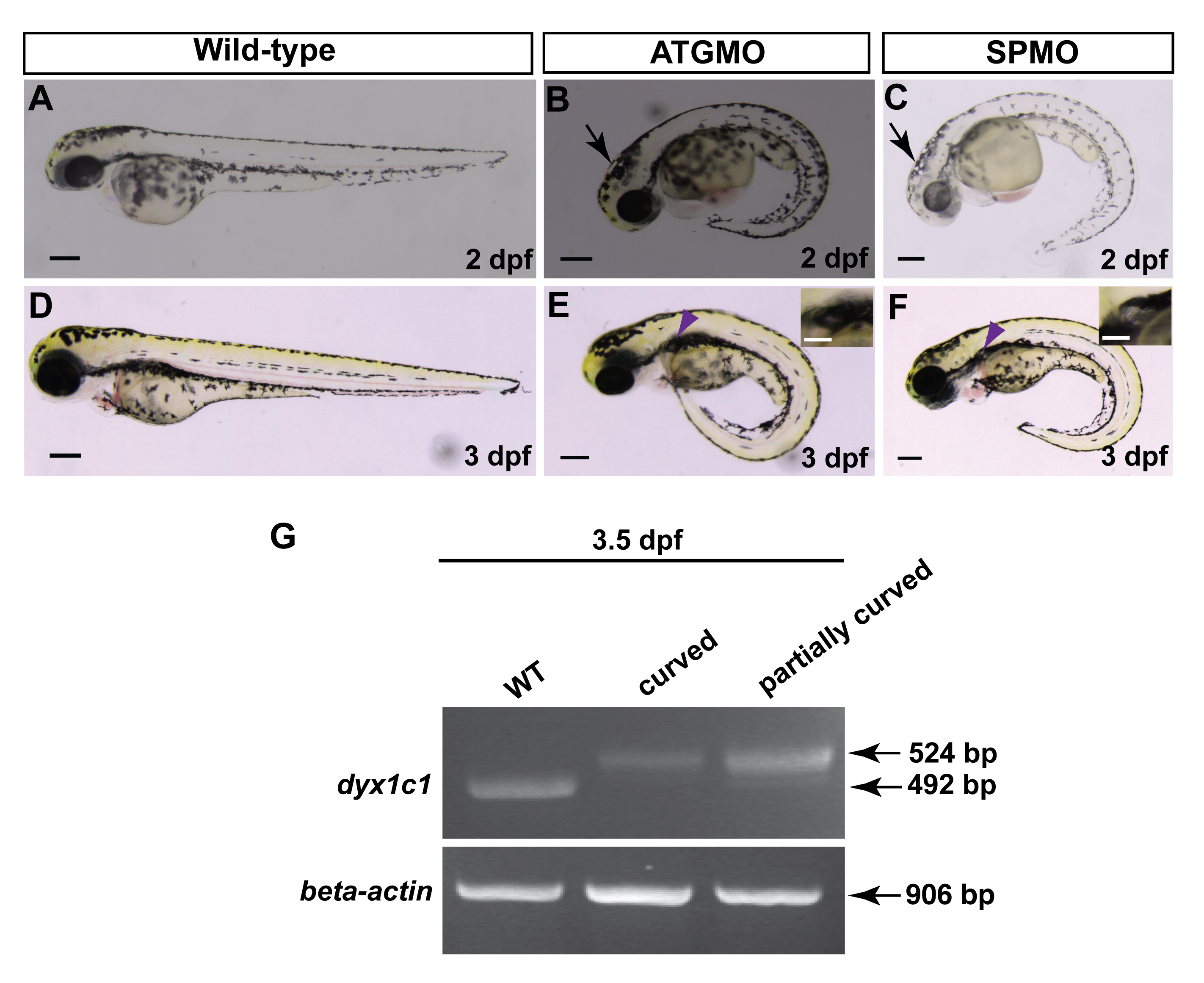

Supplement: Figure S1 — Morphological phenotypes induced by ATGMO and SPMO. Both ATGMO and SPMO when injected alone produced identical phenotypes. Hydrocephalus and kidney cysts were clearly visible in the morphants at 2 dpf (B & C) and 3 dpf (E & F) respectively as compared to the nomal phenotype in wild-type (A & D). Arrows denote hydrocephalus and kidney cysts are denoted by arrowheads. RT-PCR showing the efficiency of SPMO at 3.5 dpf in embryos showing strong and weak phenotypes (G). Scale bars indicate 100 µm. (TIF) [file pone.0063123.s001.tif]

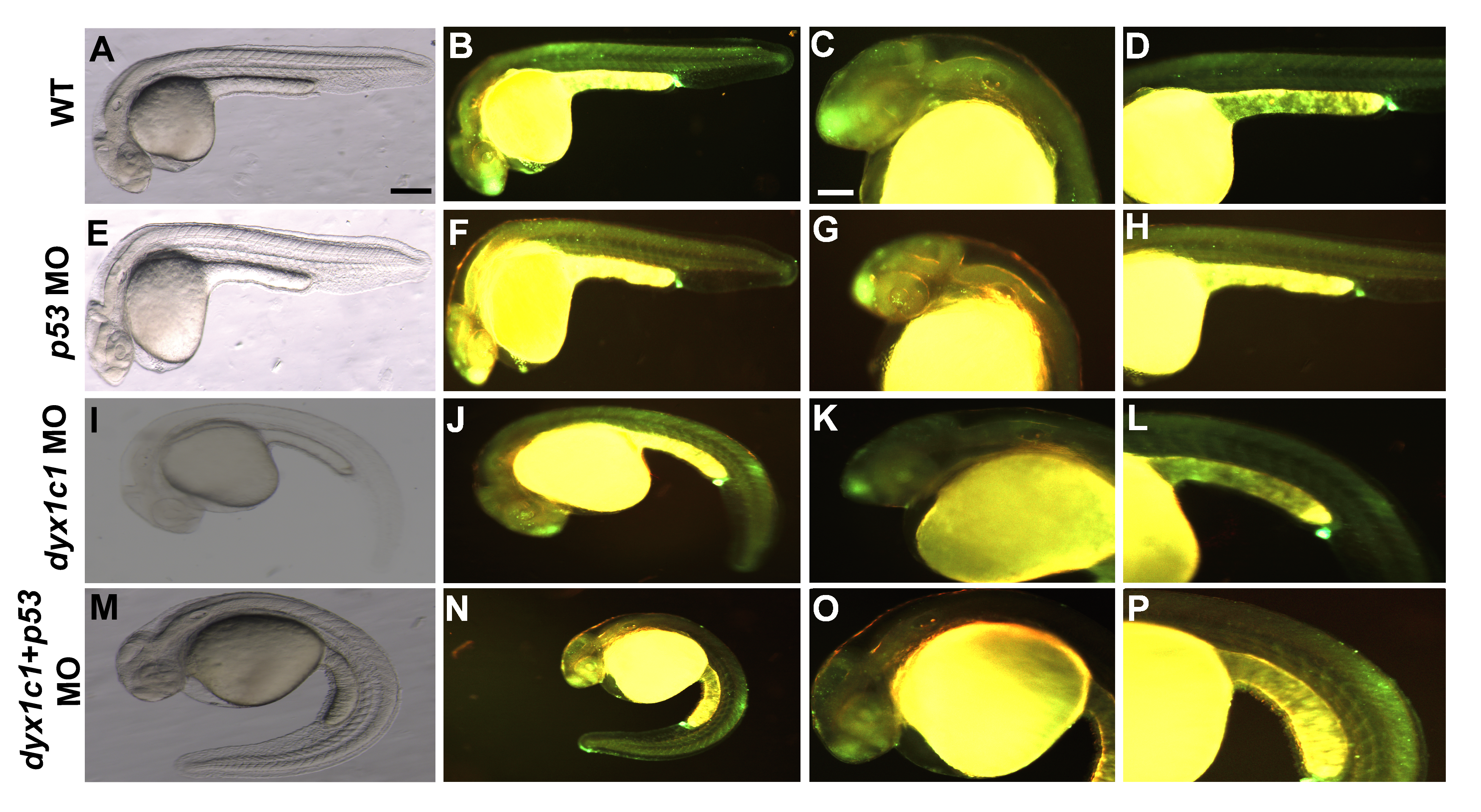

Supplement: Figure S2 — Morpholino specificity confirmed by coinjection with p53. Analysis of apoptotic cell death in WT (A–D), p53 morphants (E–H), dyx1c1 morphants (ATGMO+SPMO; I–L) and dyx1c1+ p53 morphants (M–P). Bright field (A,E,I,M) and fluorescent images of wild-type and morphants (B–D, F–H, J–L, N–P) at 1 dpf. Fluorescent signal in dyx1c1 morphants appeared similar to that seen in WT. dyx1c1 morphant phenotype was not affected by p53 coknockdown. Scale bars indicate 100 µm. (TIF) [file pone.0063123.s002.tif]

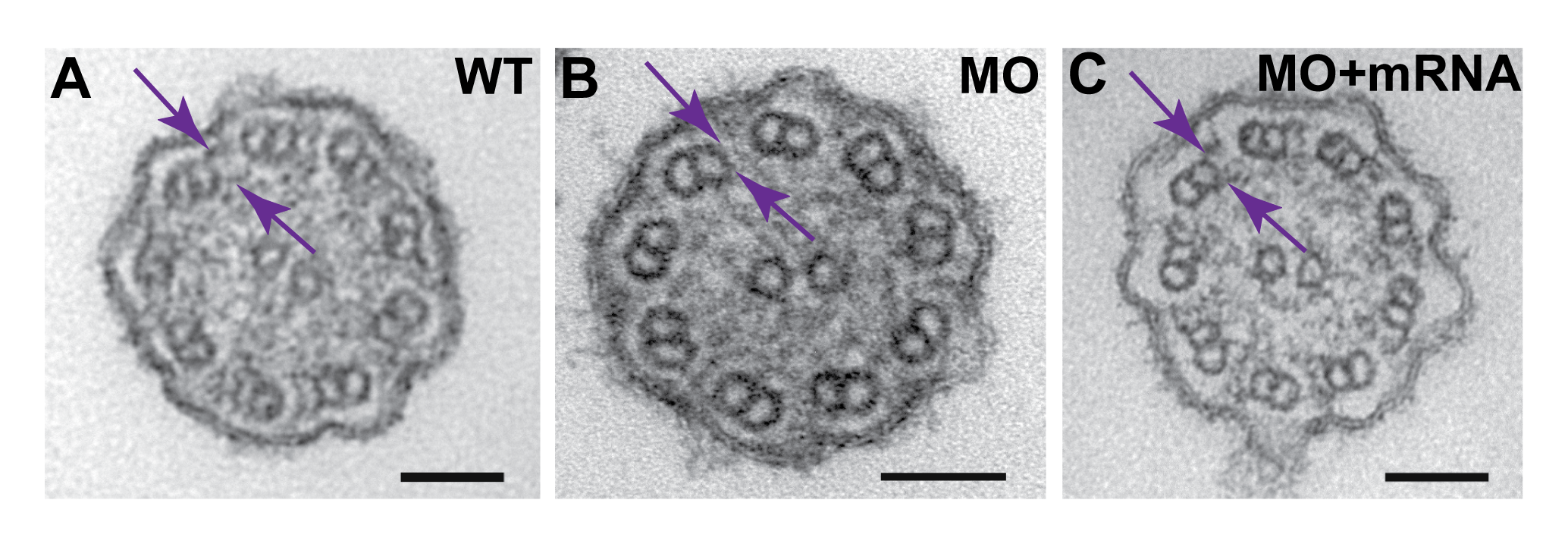

Supplement: Figure S3 — Dynein arms of olfactory cilia affected in dyx1c1 morphants. Ultrastructure of olfactory cilia at 3 dpf showed loss of ODA and IDA in dyx1c1 morphants (B) as compared to WT (A). Coinjection with dyx1c1 mRNA rescued both the dynein arms (C). Arrows denote ODA and IDA. Scale bars indicate 200 nm. (TIF) [file pone.0063123.s003.tif]

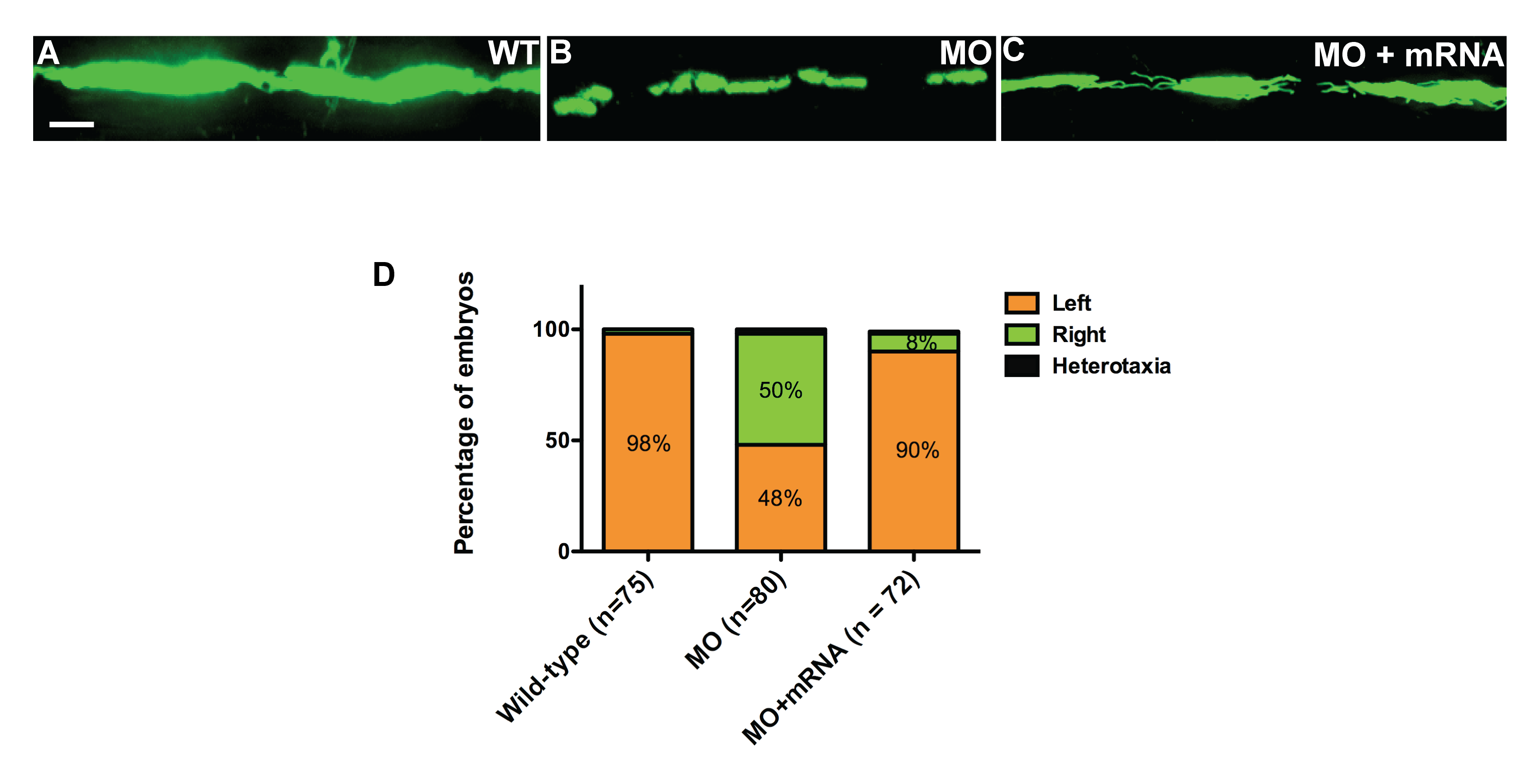

Supplement: Figure S4 — dyx1c1 mRNA rescues cilia defects in dyx1c1 morphants. Anti-acetylated tubulin staining of cilia in the pronephros of wild-type (A), dyx1c1 morphant (B) and mRNA coinjected embryo (C). Percentage of embryos showing left-side (normal), right-side (situs inversus) placement of liver and heterotaxia in wild-type, dyx1c1 morphants and mRNA injected embryos (D). Scale bar indicate 10 µm. (TIF) [file pone.0063123.s004.tif]
